# Defining the Gothic Arch Angle (GAA) as a radiographic diagnostic tool for instability in hip dysplasia

**DOI:** 10.1038/s41598-021-99011-7

**Published:** 2021-09-30

**Authors:** A. Zimmerer, J. Löchel, J. Schoon, V. Janz, G. I. Wassilew

**Affiliations:** 1 Department of Orthopedics and Orthopedic Surgery, University Medicine Greifswald, Ferdinand-Sauerbruch-Straße, 17475 Greifswald, Germany; 2 ARCUS Sportklinik Pforzheim, Rastatterstr. 17-19, 75179 Pforzheim, Germany; 3 Orthopedic Department, Center for Musculoskeletal Surgery, Charité—University Medicine, Berlin, Germany

**Keywords:** Medical research, Risk factors, Signs and symptoms

## Abstract

In recent years, there has been a controversial discussion about whether borderline dysplastic hips should be treated with an arthroscopic procedure or rather with an acetabular reorientation. New research suggests that a classification into stable and unstable hips may be helpful. The aim of the study was to validate (1) the intra- and interobserver reliability of a newly defined radiographic parameter named the Gothic Arch Angle, (2) the association between the GAA and previously existing measurements used to define severity of acetabular dysplasia, and (3) the correlation between radiographic measurements of acetabular dysplasia with MRI findings previously suggestive of hip instability. We defined and validated the GAA in 10 standardized radiographs of asymptomatic hips by two observers and calculated intra- and interobserver coefficients at two individual dates. Subsequently, a consecutive series of 100 patients with dysplastic hips (LCEA < 25°, Toennis grade ≤ 1) were evaluated for signs of instability on anteroposterior (a.p.) pelvic radiographs and direct MR arthrography and were divided in two groups: stable and unstable. In these patients the LCEA, the AI, the FEAR index and the GAA were radiographically evaluated. Correlation analyses and a logistic regression analysis was performed to identify the predictive value of instability for each radiographic parameter. Cutoff probabilities analysis was performed using standard receiver operating characteristic (ROC) curves to rate the predictive efficiency value of the GAA. The GAA showed excellent inter- and intraobserver reliability. A correlation was found between GAA and FEAR index. A logistic regression analysis showed that LCEA, FEAR index and GAA are distinct predictors of instability in hip dysplasia. The GAA showed the largest area under the curve (AUC 0.96), indicating it to be the best predictor of instability with an optimal cutoff value of 90° (sensitivity, 0.95; specificity, 0.93). The GAA is a new available indicator for instability and is thus suggested to be used as a future radiographic parameter for the stability of dysplastic hips. Further studies are needed to understand how this parameter might additionally predict clinical outcome in the treatment of hip dysplasia.

**Level of evidence:** Level III, diagnostic study.

## Introduction

Developmental dysplasia of the hip (DDH) is defined as osseous deficiency of the acetabulum with abnormal coverage of the femoral head which may lead to an overload and joint instability with subsequent damage of the acetabular cartilage or labrum^1–4^. While there is general consensus that hip dysplasia with a lateral center–edge angle (LCEA) < 18° should be treated with a reorientation of acetabular coverage through acetabular reorientation^4–8^, there is still a controversial discussion about whether milder forms of dysplasia (borderline hip) with an LCEA between 18° and 25° should be treated with arthroscopy or acetabular reorientation^3,8–14^. A few authors have suggested a distinction between stable and unstable hips for this group of patients in order to make the right therapeutic decision^11,14,15^. Wyatt et al. therefore introduced the Femoro-Epiphyseal Acetabular Roof (FEAR) index in 2017 to distinguish between stable and unstable dysplastic hips^11,15,16^. The theory underlying the FEAR index is that epiphyseal growth of the proximal femur is affected by the contact forces during development. According to Pauwels and Maquet, the epiphyseal plate orientates perpendicular to the joint reaction force in accordance with the Hueter–Volkman principle^15,17^. This leads to the fact that the angle of the closed epiphyseal plate indicates the balance of forces across the proximal femoral physis^15,18^. Wyatt et al. theorized that the angle between the epiphyseal scar of the femoral head growth plate and acetabular index (AI) would reveal the resultant force vector, which could be used to predict the stability of the borderline hip^15^. However, the originally described threshold of 5° demonstrated just a 79% probability of correctly assigning hips as stable and unstable^15^. Thus, it must be assumed that unstable hips may not be adequately identified by the FEAR index.

To integrate a broader range of biomechanical hip joint characteristics, Renato Bombelli, an Italian orthopaedic surgeon, has described the so-called gothic arch of the hip in 1976 and introduced it as the key to correct understanding of the biomechanical properties^19,20^. The gothic arch can be identified as a triangular structure on anteroposterior (ap) radiographs of the pelvis. The base of the triangle is formed by the sourcil while the sides are shaped by condensed arch-like structured trabecular bone. The intersection of these two arches form the tip of the gothic arch. Biomechanically the tip off the gothic arch lies on the line of application of the compressive force, which is perpendicular to the weight-bearing surface (WBS) and passes through the center of rotation. Bombelli hypothesized that hips with an altered gothic arch are mechanically jeopardized. In these hips the tip would therefore point either medially or laterally. In dysplastic hips the tip moves medially and thus the WBS shifts craniolaterally steeper. This in turn leads to a force directed craniolaterally, which results in increased tension on the hip capsule and consequently to craniolateral displacement of the femoral head. Consequently, we assume that the concept of the gothic arch and the FEAR Index should be combined in order to achieve a higher predictive capacity with regard to hip stability. We have therefore revisited Bombelli's concept, additionally amended it by the analysis of the femoral epiphyseal growth plate and defined a new measurement parameter for instability of the hip, called the Gothic Arch Angle (GAA).

The aims of this study were (1) to validate the intra- and interobserver reliability of a newly defined radiographic parameter named the Gothic Arch Angle, (2) to validate the association between the GAA and previously existing measurements used to define severity of acetabular dysplasia, and (3) to validate the correlation between radiographic measurements of acetabular dysplasia with MRI findings previously suggestive of hip instability.

## Methods

### Patient selection and subclassification

This is a retrospective study of prospectively collected registry data. After approval from the local ethics committee (Ethics Committee of the University of Greifswald, Germany; BB099/20) we reviewed our hospital registry to identify all patients who presented in our practice for hip joint preserving surgery between January 2019 and January 2020. From this registry, patients who showed a lateral center edge angle (LCEA) of less than 25° without signs of osteoarthritis (Toennis grade ≤ 1) on the standing a.p. pelvic radiograph and for which direct MR arthrography (dMRA) was available were selected. These patients were analyzed according to the instability criteria described by Wyatt et al.^15^ for cohort stratification. Based on these results one patient group matching stable hip criteria (n = 61) and one matching unstable hip criteria (n = 39) were formed. Both groups were compared regarding their clinical case and secondary patient characteristics (Table 1). The instability criteria described by Wyatt et al.^15^ were either an increased distance from the ilioischial line, a break of Shenton’s line, or the appearance of a crescent-shaped accumulation of gadolinium in the posteroinferior joint space at dMRA. Excluded were patients with an LCEA of more than 25° (n = 279), a missing dMRA (n = 75), osteoarthritis (Toennis grade > 1, n = 55) or previous hip surgery (n = 26). In brief, a total of 100 patients out of 535 patients could be identified and were included in the analysis (Table 1). This study has been performed in accordance with the ethical standards in the 1964 Declaration of Helsinki. All participants declared informed consent.

**Table 1:** Patient demographic data on stability groups.

	Gender	Laterality	Age, y	Body mass index, kg/m^2^
Stable (n = 61)	85% female	53% right hips	32.1 ± 12.3 (16 to 45)	23.3 ± 3.01 (16.76 to 30.78)
Unstable (n = 39)	88% female	56% right hips	31.2 ± 13.1 (17 to 43)	23.3 ± 3.01 (16.76 to 30.78)

### Measurement of the Gothic Arch Angle (GAA)

**Figure 1:**
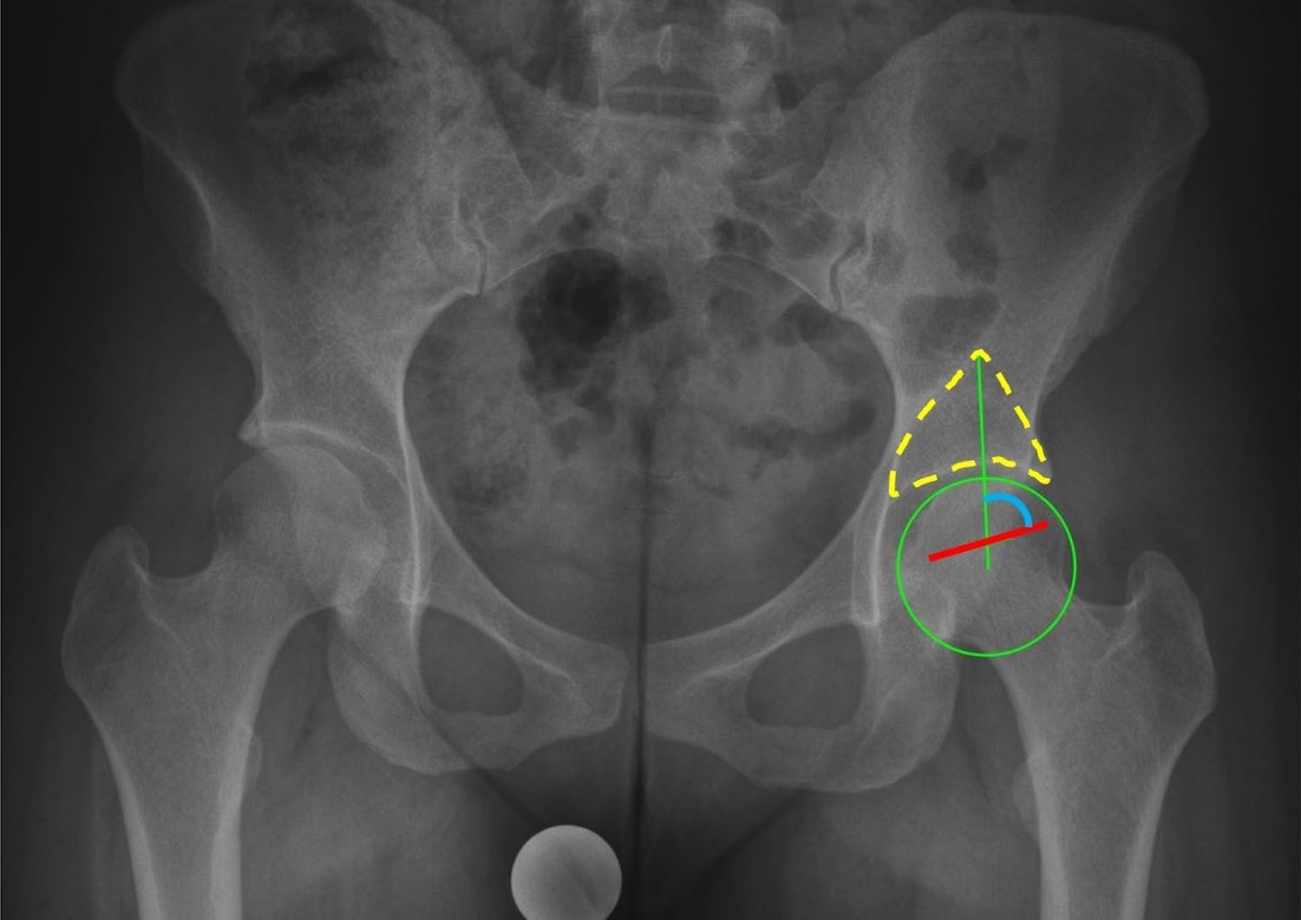
To measure the Gothic Arch Angle (blue semicircle), an angle between two lines is measured: The first line (green) extends between the center of the femoral head, which is determined by a best-fit circle around the femoral head (green circle), and the peak of the gothic arch (yellow dashed line). The second line (red) corresponds to the middle third of the physeal scar.

The GAA (Fig. 1) was measured on standing a.p. pelvic radiographs meeting the criteria to be regarded as neutrally rotated and tilted: three cm between the tip of the coccyx and the superior aspect of the symphysis pubis and symmetric obturator foramen^21,22^. An angle between two lines was measured (Fig. 1, blue semicircle). The first line (Fig. 1, green line) extends between the rotational center of the hips, which is determined by a best-fit circle around the femoral head, and the tip of the gothic arch (Fig. 1, yellow dotted-line). The base of the Gothic arch is formed by the sourcil, while the trabeculae forming the lateral aspect of the arch extend from the lateral acetabular rim toward the sacroiliac joint, while the medial aspect of the arch forms an arc of dense cancellous bone extending from the quadrilateral plate towards the anterior superior and anterior inferior iliac spines. The second line corresponds to the scar of the physeal growth plate, identical to the reference in the FEAR index^15^ (Fig. 1, red line). The central third of the scar is a straight line connecting its medial and lateral ends.

### Validation of the Gothic Arch Angle

**Figure 2:**
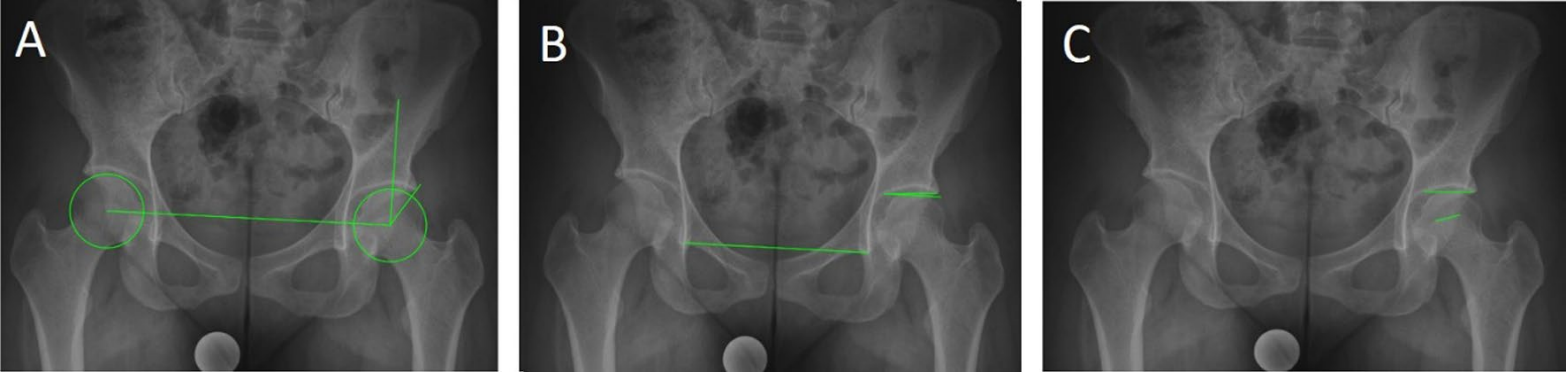
Radiographic Measurements. (**A**) Lateral Center–Edge Angle: calculated by drawing a best-fit circle around the inferior and medial margins of the femoral head. The angle is measured between 2 lines drawn from the center of the circle, one running vertically along the longitudinal axis of the pelvis and the other running along the acetabular sourcil edge^23^. (**B**) Acetabular index: measured by drawing a horizontal line parallel to the transverse pelvic axis, at the most medial edge of the sclerotic sourcil, and then making a second line extending from the medial edge to the most lateral aspect of the sourcil^24^. (**C**) Femoroepiphyseal acetabular roof index: formed by 2 lines connecting the acetabular roof inclination and the femoral head physeal scar^15^.

To validate the GAA, standardized a.p. pelvic radiographs of 20 asymptomatic patients were analyzed as previously decribed^15^. Those patients were treated for an unrelated trauma in our hospital in May 2020. In addition to the GAA, the LCEA^23^, the acetabular index (AI)^24^, and the FEAR index^15^ (Fig. 2) were measured by two independent orthopaedic surgeons (A.Z., J.L.) using the software mediCAD (mediCAD Hectec GmbH, Altdorf, Germany).

### Data presentation and statistics

Continuous variables are presented as means ± SD with ranges, and categorical variables are presented as frequencies and percentages. Data were analyzed by use of R (R Core Team, 2017)^25^. Statistical reliability testing of the radiological parameters was performed with intraclass correlation values interpreted as: greater than 0.75 = excellent, 0.40–0.75 = fair to good, and less than 0.40 = poor^26,27^. Bland–Altman graphs were generated to present interobserver agreement^28^. The Spearman coefficient analysis was used to identify correlations between the radiological measurements. Correlation coefficients were classified by the strength of the correlation: excellent (> 0.80), very good (0.71–0.80), good (0.61–0.70), fair (0.41–0.60), and poor (0.21–0.40). A stepwise forward multivariate logistic regression analysis was performed to identify the predictive value of an instability for each radiographic measurement. Cutoff probabilities and sensitivity/specificity analysis were performed using standard receiver operating characteristic (ROC) curves to determine optimal cutoff values. The threshold for statistical significance was set to 0.05.

## Results

### Validation of the Gothic Arch Angle

**Figure 3:**
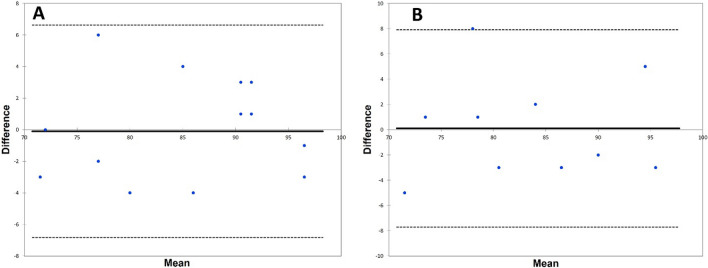
Bland Altmann plots for the GAA. (**A**) First measurement. (**B**) Second measurement.

Two observers (A.Z., J.L.) independently measured the GAA. Each image was measured at two independent time points (one week apart). The GAA showed excellent inter- and intraobserver agreement. In addition, the reliability of the GAA was compared with the LCEA, the AI, and the FEAR index (Table 2). The measured GAA did not differ significantly between the examiners and date of review (Fig. 3).

**Table 2:** Intra- and Interobserver Reproducibility of the GAA in 20 Patients^a^.

	Intraobserver reproducibility (Observer 1)	Intraobserver reproducibility (Observer 2)	Interobserver reproducibility
	ICC (95% CI)	ICC (95% CI)	ICC (95% CI)
LCEA	0.918 (0.867–0.976)	0.872 (0.832–0.987)	0.910 (0.835–0.965)
AI	0.913 (0.875–0.983)	0.921 (0.876–0.985)	0.916 (0.845–0.975)
FEAR Index	0.994 (0.982–0.999)	0.962 (0.863–0.992)	0.983 (0.893–0.996)
GAA	0.996 (0.994–1.0)	0.995 (0.907–0.994)	0.957 (0.890–0.988)

^a^*AI* acetabular index, *FEAR index* Femoro-Epiphyseal Acetabular Roof, *GAA* Gothic Arch Angle, *ICC* intraclass correlation coefficient, *LCEA* Lateral Center–Edge Angle;

### Correlation analysis

**Figure 4:**
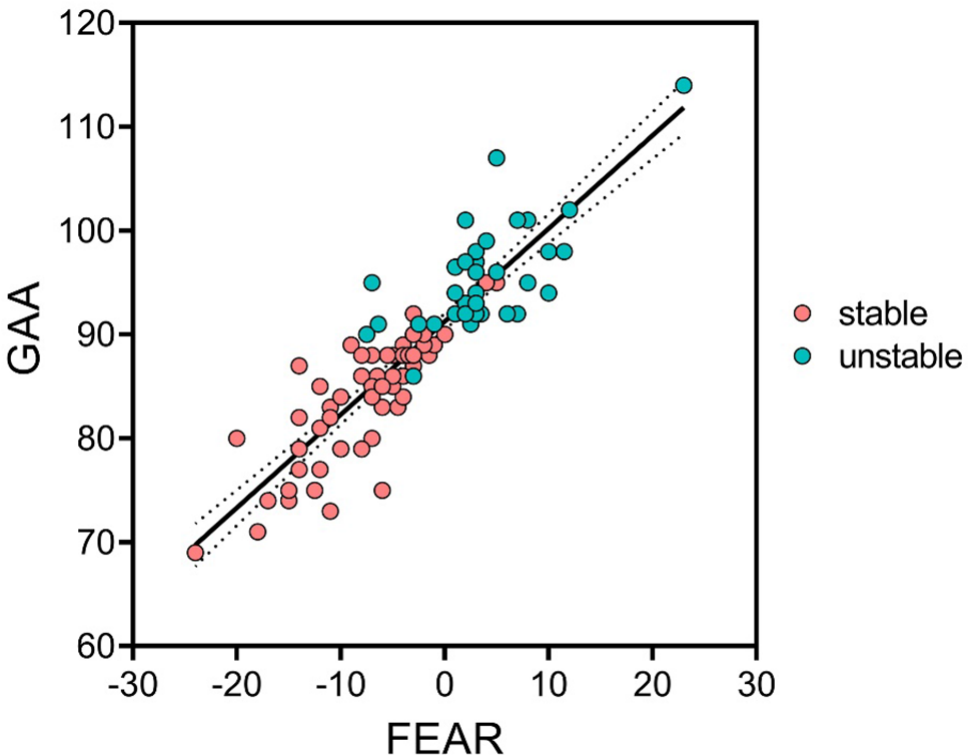
Scatter plot of the relation between GAA and FEAR index for dysplastic hips (LCEA < 25°). Stable hips are displayed in red, unstable hips in green; *GAA* Gothic Arch Angle, *FEAR Index* Femoro-Epiphyseal Acetabular Roof Index.

After validation of the GAA, a correlation analysis between LCEA, AI, FEAR index and GAA was performed (Table 3). An excellent correlation was found between the GAA and the FEAR index (r = 0.899, p < 0.0001) (Fig. 4).

**Table 3:** Spearman correlation coefficients of the radiological measurements for all included hips^a^.

Variables	LCEA	AI	FEAR Index	GAA
LCEA	1	**− 0.306**	**− 0.316**	**− 0.321**
AI	**− 0.306**	1	**0.299**	0.068
FEAR index	**− 0.316**	**0.299**	1	**0.899**
GAA	**− 0.321**	0.068	**0.889**	1

^a^Bold values are statistically significant.

*AI* acetabular index, *GAA* Gothic Arch Angle, *FEAR Index* Femoro-Epiphyseal Acetabular Roof Index, *LCEA* Lateral Center–Edge Angle.

### Assessment of Radiographic Parameters

**Figure 5:**
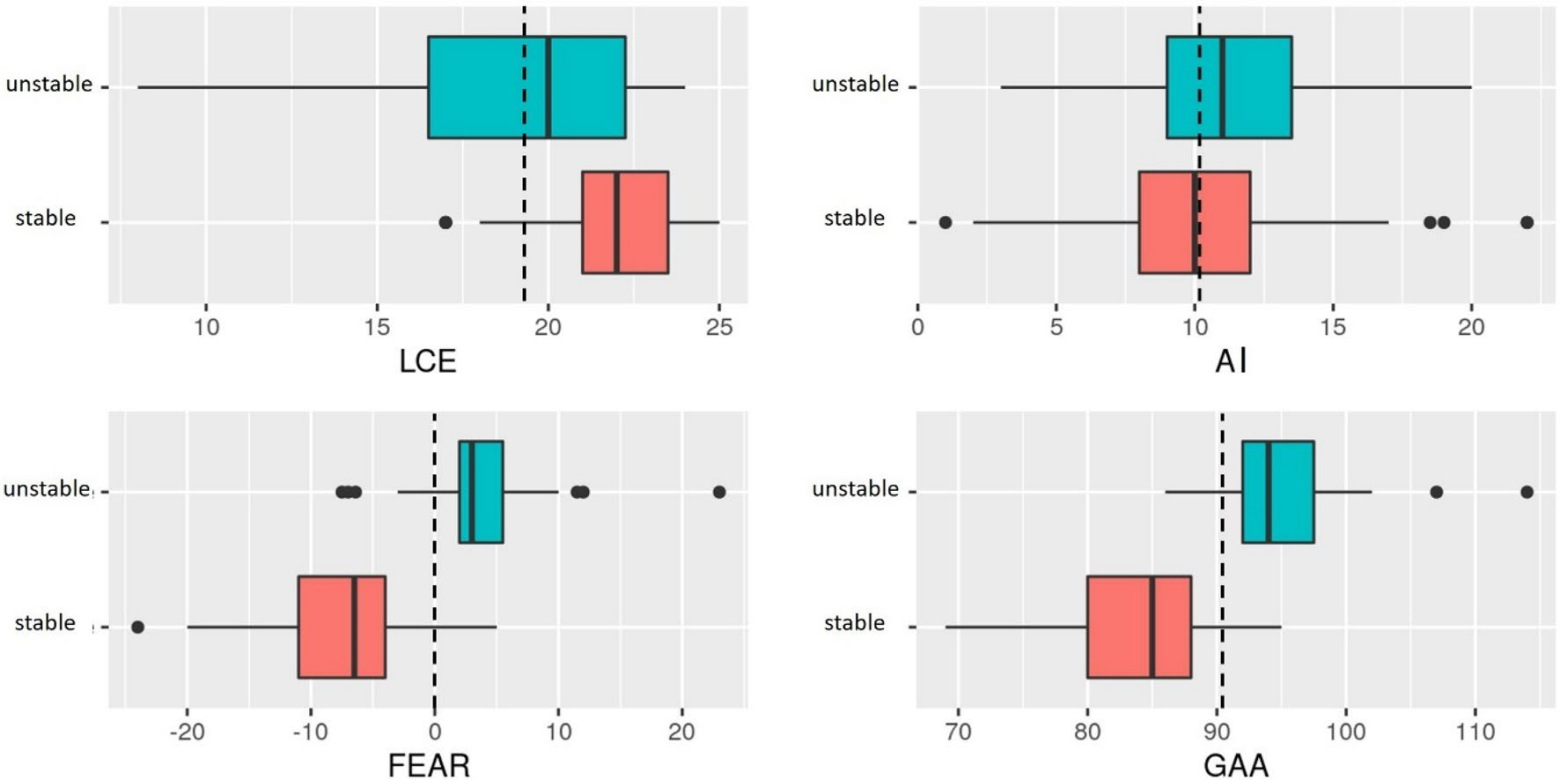
Boxplots of the radiographic parameters for the two groups. The dashed lines indicate the optimal cutoff values for predicting instability. It is obvious that LCEA and AC angle are not suitable to prediction of instability.

All radiographic parameters were significantly different between the stable and unstable hips, except for AI (10.5° vs. 10.9; p = 0.578) (Table 4 and Fig. 5). The LCEA was found to be smaller in the unstable group (21.8° vs. 19.1; p < 0.001). The FEAR index (− 7.5° vs. 3.5°; p < 0.0001) and GAA (84.0° vs 95.2°; p < 0.0001) for patients meeting criteria for instability were significantly more positive.

**Table 4:** Radiographic measurements by stability diagnosis^a^.

	Stable hip	Unstable hips	*P* Value
LCEA, deg	21.8 ± 2.1 (17–25)	19.1 ± 4.0 (8–24)	< 0.001
AI, deg	10.5 ± 4.2 (1–22)	10.9 ± 3.5 (2–20)	0.578
FEAR index, deg	− 7.5 ± 5.6 (− 24–5)	3.5 ± 5.4 (− 7.5–23)	< 0.0001
GAA, deg	84.0 ± 5.8 (69–95)	95.2 ± 5.0 (86–114)	< 0.0001

^a^Data are shown as mean ± SD (range).

*AI* acetabular index, *GAA* Gothic Arch Angle, *FEAR Index* Femoro-Epiphyseal Acetabular Roof Index, *LCEA* Lateral Center–Edge Angle.

The logistic regression analysis showed that the LCEA (p < 0.001), FEAR index (p < 0.0001) and GAA (p < 0.0001) were significantly different between the unstable and stable patient group, while the AI (p = 0.59) did not indicate significant differences (Table 5).

**Table 5:** Logistic regression analysis of radiographic parameters for instability^a^.

	*P* Value
LCEA	< 0.001
AI	0.59
FEAR index	< 0.0001
GAA	< 0.0001

^a^*AI* acetabular index, *GAA* Gothic Arch Angle, *FEAR Index* Femoro-Epiphyseal Acetabular Roof Index, *LCEA* Lateral Center–Edge Angle.

**Figure 6:**
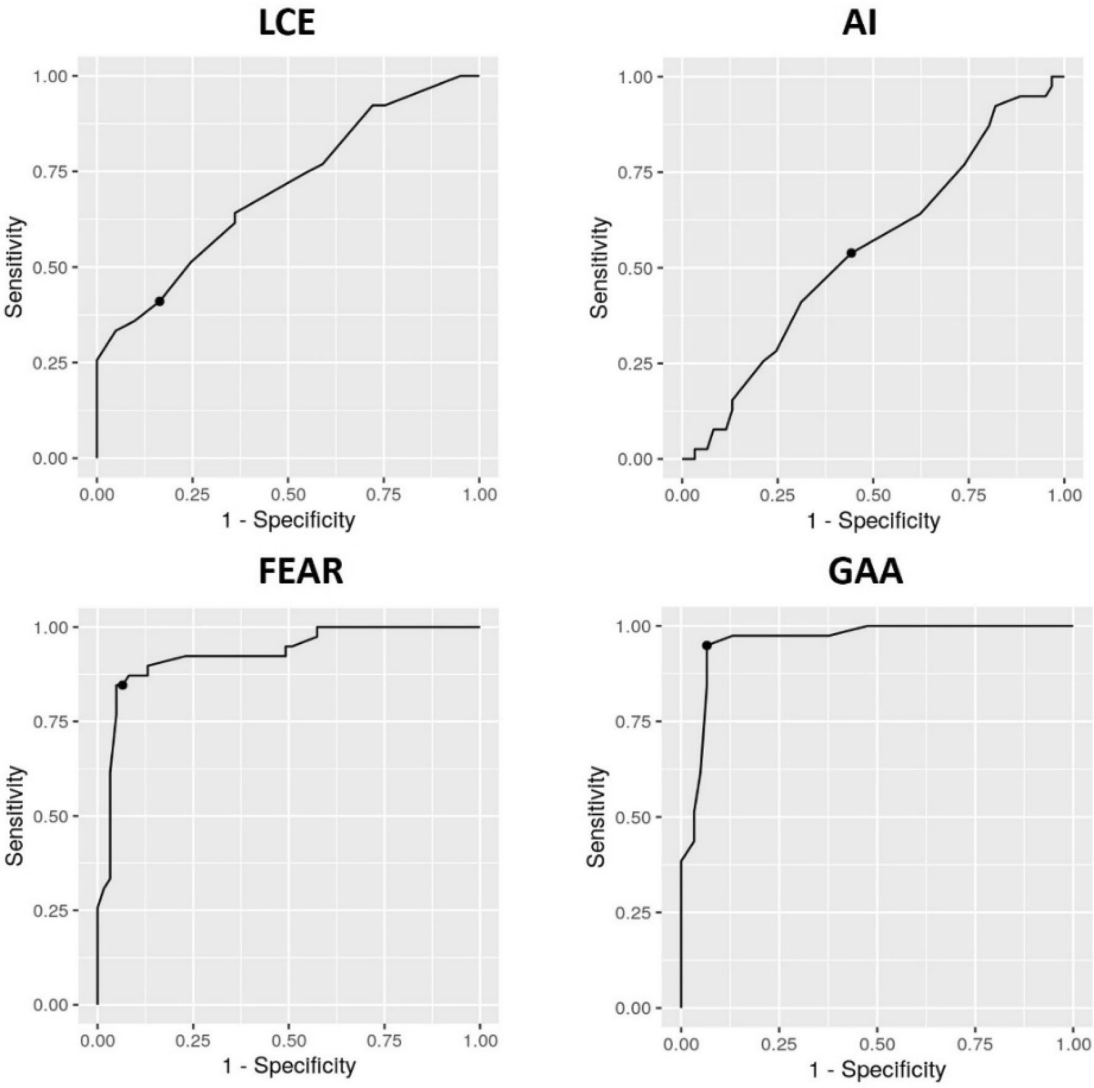
Simultaneous receiver operating characteristic (ROC) curves for radiographic parameters. *AI* acetabular index, *FEAR* Femoro-epiphyseal Acetabular Roof, *GAA* Gothic Arch Angle, *LCEA* Lateral Center Edge Angle.

To compare the four radiographic parameters in terms of their predictive efficiency for instability, we plotted simultaneous ROC curves, which are shown in Fig. 6. The GAA had the largest area under the curve (AUC 0.96), indicating its superior predictive efficiency for instability. We found the optimal cutoff value for FEAR index at 0° (sensitivity, 0.84; specificity, 0.93) and for GAA at 90° (sensitivity, 0.95; specificity, 0.93), respectively.

## Discussion

In the present study we have defined a new radiological parameter, which is intended to be a predictor for instability in dysplastic hips. We could demonstrate excellent intra- and interobserver reliabilities for this new radiological parameter. Furthermore, the GAA was identified as the most effective indicator of radiologically assessed instability. The optimal GAA cutoff value for the differentiation between stable and unstable hips was 90°. More precisely, hips with a GAA > 90° can be considered unstable and vice versa.

In recent years, there has been increasing discussion about whether so-called borderline dysplastic hips should be treated with an arthroscopic procedure or rather with an acetabular reorientation. Arthroscopic interventions for the management of borderline dysplastic hips have been described with varying degrees of success and high rates of re-operation and conversion to total hip arthroplasty^3,8–10,13,14,29–32^. In contrast, good results and minimal complications were reported in patients treated with PAO after 1 year and 2 years postoperatively^10,32^. The unpredictability of the results, especially with arthroscopic treatment, can be due to difficulty in differentiating between stable and unstable hips. In this context, much of the literature has isolated the definition of dysplastic hips to the LCEA evaluation, which is a reliable measure of lateral acetabular coverage, but not a surrogate for the global acetabular morphology. The sole determination of the LCEA can mischaracterize the nature of acetabular under-coverage and it could be shown that an adequate classification should be obtained by several radiological parameters^8,10,12,33,34^.

However, we believe, similar to Wyatt et al.^15^, that a classification into borderline and severe dysplasia is obsolete. Rather, a functional analysis should be performed in order to be able to make a valid classification. In our opinion, the challenge is to classify the hip as either stable or unstable and subsequently treat it in the appropriate manner. Wyatt et al. introduced the FEAR index^15^ to differentiate between stable and unstable hips, which can also be applied as an indicator for micro-instabilities in non-dysplastic hips^35^. Thus, the originally described threshold of 5° demonstrated just a 79% probability of correctly assigning hips as stable and unstable^15^. Another conception that allows the evaluation of hip stability is the acetabular gothic arch described by Bombelli in 1976^19,20^. The gothic arch represents a characteristic feature of anteroposterior radiographs of the pelvis. Bombelli hypothesized that hips with an abnormal Gothic arch are mechanically compromised and predisposed to the development of osteoarthritis. In normal hips, the apex of the Gothic arch lies directly over the center of the femoral head, so that a line connecting these points is exactly perpendicular. In abnormal hips, however, the apex of the Gothic arch lies medial or lateral to a vertical line drawn through the center of the femoral head, resulting in a craniomedial or craniolateral orientation of the Gothic arch. However, Bombelli did not define cut-off values. We assumed that a combination of both concepts may allow an increased prediction regarding stability. Therefore, we have adopted both concepts and combined them with the gothic arch angle to a holistic approach and analysis of hip geometry in order to provide the most accurate possible prediction of stability. We were able to define a threshold of 90°, which demonstrated high sensitivity and specificity (sensitivity, 0.95; specificity, 0.93) with respect to discrimination into stable or unstable. Based on the results of our study, it may be feasible for clinicians to use thee GAA as an additional radiologic assessment to identify patients with possible abnormal mechanics leading to hip instability.

In our study, the AI was not found to predict hip instability. This may be due to the fact that only a few hips with increased AI are underrepresented in the present analysis. However, the AI is included in the determination of the FEAR index, which was found to be a significant predictor of instability. It can therefore be assumed that the orientation and position of the epiphyseal scar is the more important factor in the FEAR index. Bombelli proposed the theory that a craniomedially aligned tip of the gothic arch must be considered an indication of hip dysplasia, which has been confirmed in the past^36^. Herickhoff et al. demonstrated in patients with unilateral DDH that the tip of the gothic arch pointed significantly more medially on the dysplastic side than on the healthy side (4.43° [Normal Hip] vs. 15.33 [DDH Hip]; p = 0.0001)^36^. We are convinced that the presented concept allows an even more reliable prediction of instability when both aspects (FEAR Index and Gothic Arch) are combined.

The values for the FEAR index and GAA were significantly higher in the unstable group than in the stable group. Compared to the FEAR index, however, the GAA demonstrated the highest AUC in the ROC analysis and therefore appears to be a better predictor of instability. However, the GAA should not be used as the solely tool, but rather as a further piece in the puzzle on the way to a reliable diagnosis and identification of unstable hips. In recent years, a relationship between femoral torsion and dysplasia has been observed, thus the femoral torsion dimension should be considered in an appropriate assessment^37,38^. We therefore recommend a standardized approach, which includes patient history and examination as well as diagnostics consisting of radiography and MRI. The GAA can provide significant information regarding hip stability. However, the impact of the GAA on clinical outcomes needs to be demonstrated in further studies.

### Limitations

All patients had symptomatic hip pathologies, hence the study lacks an asymptomatic control group. There is no correlation with clinical results that are based on a therapy decision derived from the use of GAA. Future studies investigating the influence of the GAA on clinical results are therefore mandatory. A further limitation, although it did not apply to any of the radiographs analyzed, is the possibility that the epiphyseal scar or the tip of the gothic arch cannot be identified due to radiograph quality. In such cases it may theoretically not be possible to determine the GAA. However, this limitation also applies to the FEAR index. Furthermore, there is no data on whether the epiphyseal scar changes or remains consistent over time or whether the femoral version has an influence on the assessment of the GAA. Finally, the GAA is evaluated by means of static radiographic images, so that no dynamic assessment and analysis is possible. Prospective studies may be conducted to examine the dynamic motion and the influence on stability.

## Conclusion

We defined a new radiological parameter to assess the stability of dysplastic hips. The GAA demonstrated excellent intra- and interobserver reliabilities. The optimal cutoff value for the GAA was 90° to differentiate between stable and unstable hips. Further studies are needed to understand how this parameter might additionally predict clinical outcome in the treatment of symptomatic hip dysplasia.
