# Author Correction: Defining the Gothic Arch Angle (GAA) as a radiographic diagnostic tool for instability in hip dysplasia

**DOI:** 10.1038/s41598-021-00095-y

**Published:** 2021-10-13

**Authors:** A. Zimmerer, J. Löchel, J. Schoon, V. Janz, G. I. Wassilew

**Affiliations:** 1 Department of Orthopedics and Orthopedic Surgery, University Medicine Greifswald, Ferdinand-Sauerbruch-Straße, 17475 Greifswald, Germany; 2 ARCUS Sportklinik Pforzheim, Rastatterstr. 17-19, 75179 Pforzheim, Germany; 3 Orthopedic Department, Center for Musculoskeletal Surgery, Charité—University Medicine, Berlin, Germany

Correction to: *Scientific Reports* 10.1038/s41598-021-99011-7, published online 30 September 2021

The original version of this Article contained an error in the Abstract and Introduction, where

“Gothic”

now reads:

“Gothic Arch Angle”

As the result, in the Abstract,

“The aim of the study was to validate (1) the intra- and interobserver reliability of a newly defined radiographic parameter named the Gothic, (2) the association between the GAA and previously existing measurements used to define severity of acetabular dysplasia, and (3) the correlation between radiographic measurements of acetabular dysplasia with MRI findings previously suggestive of hip instability.”

now reads:

“The aim of the study was to validate (1) the intra- and interobserver reliability of a newly defined radiographic parameter named the Gothic Arch Angle, (2) the association between the GAA and previously existing measurements used to define severity of acetabular dysplasia, and (3) the correlation between radiographic measurements of acetabular dysplasia with MRI findings previously suggestive of hip instability.”

In the Introduction,

“The aims of this study were (1) to validate the intra- and interobserver reliability of a newly defined radiographic parameter named the Gothic, (2) to validate the association between the GAA and previously existing measurements used to define severity of acetabular dysplasia, and (3) to validate the correlation between radiographic measurements of acetabular dysplasia with MRI findings previously suggestive of hip instability.”

now reads:

“The aims of this study were (1) to validate the intra- and interobserver reliability of a newly defined radiographic parameter named the Gothic Arch Angle, (2) to validate the association between the GAA and previously existing measurements used to define severity of acetabular dysplasia, and (3) to validate the correlation between radiographic measurements of acetabular dysplasia with MRI findings previously suggestive of hip instability.”

Additionally, in the Methods, under the subheading ‘Validation of the Gothic Arch Angle’,

“In addition to the GAA, the LCEA^23^, the acetabular index (AI)^24^, and the FEAR index^15^ (Fig. 2) were measured by two independent orthopaedic surgeons (X.X., Y.Y.) using the software mediCAD (mediCAD Hectec GmbH, Altdorf, Germany).”

now reads:

“In addition to the GAA, the LCEA^23^, the acetabular index (AI)^24^, and the FEAR index^15^ (Fig. 2) were measured by two independent orthopaedic surgeons (A.Z., J.L.) using the software mediCAD (mediCAD Hectec GmbH, Altdorf, Germany).”

And, in the Results section, under the subheading ‘Validation of the Gothic Arch Angle’,

“Two observers (X.X., Y.Y.) independently measured the GAA.”

now reads:

“Two observers (A.Z., J.L.) independently measured the GAA.”

The original Article has been corrected.

